# High CD3+ Cells in Intracranial Thrombi Represent a Biomarker of Atherothrombotic Stroke

**DOI:** 10.1371/journal.pone.0154945

**Published:** 2016-05-06

**Authors:** Cyril Dargazanli, Valérie Rigau, Omer Eker, Carlos Riquelme Bareiro, Paolo Machi, Grégory Gascou, Caroline Arquizan, Xavier Ayrignac, Isabelle Mourand, Astrid Corlobé, Kyriakos Lobotesis, Nicolas Molinari, Valérie Costes, Alain Bonafé, Vincent Costalat

**Affiliations:** 1 Department of Neuroradiology, Montpellier University Hospital Center, Gui de Chauliac Hospital, Montpellier, France; 2 Department of Pathology, Montpellier University Hospital Center, Gui de Chauliac Hospital, Montpellier, France; 3 Department of Neurology, Montpellier University Hospital Center, Gui de Chauliac Hospital, Montpellier, France; 4 Department of Imaging, Imperial College Healthcare NHS Trust, Charing Cross Hospital, London, United Kingdom; 5 IMAG UMR 5149, University of Montpellier, School of Pharmacy, Montpellier University Hospital Center, Colombière Hospital, Montpellier, France; Nagoya University, JAPAN

## Abstract

**Background and Purpose:**

Approximately 30% of strokes are cryptogenic despite an exhaustive in-hospital work-up. Analysis of clot composition following endovascular treatment could provide insight into stroke etiology. T-cells already have been shown to be a major component of vulnerable atherosclerotic carotid lesions. We therefore hypothesize that T-cell content in intracranial thrombi may also be a biomarker of atherothrombotic origin.

**Materials and Methods:**

We histopathologically investigated 54 consecutive thrombi retrieved after mechanical thrombectomy in acute stroke patients. First, thrombi were classified as fibrin-dominant, erythrocyte-dominant or mixed pattern. We then performed quantitative analysis of CD3+ cells on immunohistochemically-stained thrombi and compared T-cell content between “atherothrombotic”, “cardioembolism” and “other causes” stroke subtypes.

**Results:**

Fourteen (26%) thrombi were defined as fibrin-dominant, 15 (28%) as erythrocyte-dominant, 25 (46%) as mixed. The stroke cause was defined as “atherothrombotic” in 10 (18.5%), “cardioembolism” in 25 (46.3%), and “other causes” in 19 (35.2%). Number of T-cells was significantly higher in thrombi from the “atherothrombotic” group (53.60 ± 28.78) than in the other causes (21.77 ± 18.31; p<0.0005) or the “cardioembolism” group (20.08 ± 15.66; p<0.0003).

**Conclusions:**

The CD3+ T-cell count in intracranial thrombi was significantly higher in “atherothrombotic” origin strokes compared to all other causes. Thrombi with high content of CD3+ cells are more likely to originate from an atherosclerotic plaque.

## Introduction

Stroke is a significant public health problem, with an incidence and a prevalence of 9 and 30.7 million respectively, according to the World Health Organization. It is a heterogeneous disease, with more than 150 causes identified [1]. Thromboembolic occlusion is the main etiology, and is mostly caused by large artery disease (i.e. internal carotid artery atherosclerosis, arterial dissections) and cardioembolism.

Up to now, cerebral thrombi were inaccessible and intravenous thrombolysis with recombinant tissue plasminogen activator (rtPA) was the only treatment available [2]. Recent advances in endovascular treatment, specifically stent retrievers and aspiration [3] allows retrieval of cerebral thrombi from brain arteries, and subsequently their systematic histopathologic analysis. Few studies on intracranial thrombi have been undertaken to date. They mainly reported variable architecture of cerebral thrombi with different components such as fibrin, red blood cells (RBC) and platelets [4,5]. No advanced subtype classification of blood components were performed. Nevertheless, studying histopathologic clot composition of intracranial thrombi could potentially have high clinical relevance. Firstly, a significant proportion (30%) of strokes are cryptogenic despite an exhaustive work-up [6]. Secondly, clot composition may influence the efficacy of fibrinolysis [7] or affect the success rate of endovascular intervention [8].

As secondary prevention is based on the stroke etiology, we may assume that one third of the patients receive only a probabilistic medication, not necessarily matching accurately their etiology.

Only three studies analyzed white blood cells in the intracranial thrombi, but without differentiating their subtypes [4,9,10]. Immunochemistry allows precise analysis of white cells subtypes, already identified as potential biomarkers of atheromatous vulnerable lesion [11].

T-cells already have been shown to be a major component of vulnerable atherosclerotic carotid lesions [12]. The aim of our study was to relate histopathologic composition of thrombi in terms of T-cells to the stroke etiology, using immunochemistry with anti-CD3 antibodies. The T-cell antigen receptor (TCR), present on each T-cell, consists of a glycoprotein heterodimer, which is non-covalently associated with at least four or five invariant CD3 proteins. Thus, CD3 is considered as a pan T-cell antigen. The main hypothesis of this study was that CD3+ cell content inside intracranial thrombi can assist in screening for an atherothrombotic source stroke, with the final goal being the utilization of immunochemistry on intracranial thrombi as an etiological biomarker.

## Materials and Methods

### Study design

This prospective study was conducted at the Gui de Chauliac University Hospital in Montpellier, France.

Consecutive patients admitted in our center with acute cerebral ischemic infarct were enrolled from January 2014 to September 2014.

The study was approved by the local ethics committee (Comité de Protection des Personnes «Sud-Méditerranée IV», Centre Hospitalier Universitaire de Montpellier, hôpital Saint-Eloi, 34295 Montpellier Cedex 5), with the patients providing written and informed consent in acute phase when possible. Otherwise, the consent form was signed by the patient’s relatives. All procedures were in accordance with institutional guidelines and protocols [13]. All patients eligible for an endovascular treatment of ischemic stroke were included: acute ischemic stroke with proximal arterial occlusion (cervical or terminal carotid artery, first or second segment of the middle cerebral artery, and basilar artery), harboring a significant clinical impairment (NIHSS ≥8), and a limited ischemic core volume (DWI-ASPECT score ≥5).

Patients with a thromboembolic material unsuitable for histopathologic examination (procedure failure, thromboembolic material too small) were excluded.

The following baseline data were collected: sex, age, cardiovascular risk factors (hypertension, diabetes mellitus, smoking), NIHSS at presentation, and location of the thrombus (cervical carotid, carotid T, middle cerebral artery, and basilar artery).

Stroke etiology was defined as “atherosclerotic” when CTA or MRA demonstrated significant (50%) stenosis or occlusion of a major cervical (carotid or vertebral) artery or intracranial artery ipsilateral to the symptomatic hemisphere, presumed to be due to atherosclerosis according to the Trial of Org 10172 in Acute Stroke Treatment (TOAST) criteria [14], in adjunction to exclusion of potential sources of cardiac embolism. Stroke etiology was defined as “cardioembolism” when at least one cardiac source for an embolus was identified after a complete cardiologic work-up including Holter monitoring and echocardiography, in the absence of significant stenosis (50%) of ipsilateral large extracranial arteries or significant atherosclerosis. Stroke etiology was defined “dissection” when a narrowed eccentric lumen surrounded by crescent-shaped mural thickening or tapered occlusion with associated increase in external vessel diameter on CTA or MRI was shown, with angiographic confirmation. Stroke etiology was defined as “other causes” when intracranial stenosis, thrombophilia (on the basis of both clinical and biological data), endocarditis, and peri-procedural thromboembolic complication during endovascular treatment occurred. Stroke etiology was defined as “unknown” when no reliable etiology was found after a complete clinical, laboratory and imaging work-up. Stroke etiology was defined as “undefined” when at least two etiologies were found after a complete clinical, laboratory and imaging work-up (for example atrial fibrillation and permeable foramen ovale).

### Intravenous fibrinolysis

Intravenous thrombolysis with recombinant human tissue plasminogen activator (0.9 mg/kg) was considered in all patients presenting within 4.5 hours from symptom onset. A loading dose of 10% was administered as soon as possible. Patient contraindications for IV fibrinolysis were: pregnancy, serum glucose <50 and >400 mg/dl, known hemorrhagic diathesis, known coagulation factor deficiency, oral anticoagulation treatment with international normalized ratio >1.7, use of heparin with a prothrombin time >2-times normal, platelet count <100000/ml, sustained systolic blood pressure >185 mmHg or diastolic blood pressure >110 mmHg despite intravenous treatment, and life expectancy <3 months.

### Thrombectomy procedure

All procedures were performed via a femoral artery approach under general anesthesia (GA). In the anterior circulation, an 8-Fr or 9-Fr balloon guide catheter (Merci Balloon Guide Catheter; Concentric Medical) was introduced through a femoral sheath into the concerned carotid artery. In the posterior circulation, a 6-Fr Envoy (Cordis) guide catheter was placed into the dominant or most navigable vertebral artery. A 0.21-inch internal diameter microcatheter (Prowler Select Plus; Cordis or Vasco 21; Balt) was navigated distal to the point of occlusion over a 0.014-inch steerable microwire. The microwire was exchanged with the mechanical thrombectomy device. All patients undergoing mechanical thrombectomy were treated using the Solitaire FR or AB device (Covidien/Medtronic). An intravenous bolus of 1000 UI of standard heparin was administered after Solitaire FR device deployment. On Solitaire FR device deployment, an angiographic run was performed to evaluate the correct placement and expansion of the device. The device was left in place for 2 to 7 minutes, allowing full expansion of the nitinol stent through the thrombus. Then, the fully deployed Solitaire FR device together with the delivery microcatheter were gently pulled back as a single unit and recovered. Before this retrieval, the balloon guide catheter was inflated and manual aspiration was performed with a 50 ml syringe through the hemostatic valve to reverse the flow in the target artery and therefore reduce the risk of thromboembolism. Successful recanalization was defined as Thrombolysis In Cerebral Infarction (TICI) 3 in all treatable vessels. If the treatable vessel was not opened to at least TICI 2 with a maximum of 5 passes of the thrombectomy device, then the treatment was considered as a failure. No intra-arterial fibrinolytics were administered at any point during this study, even if the recanalization attempt was unsuccessful. Groin punctures were routinely closed with an Angio-Seal (St. Jude Medical, St. Paul, MN).

### Orientation of the thrombus

Orientation of the thrombus, once gently retrieved from the stent, was maintained if possible. The proximal part in contact with blood flow was marked in green by a special tattoo paste (Green tattoo ink paste, Santa Cruz Biotechnology), then thrombi were deposited in physiological saline at 37°C and directly sent to the pathology department.

### Histopathologic procedure

Formalin-fixed specimens were embedded in paraffin and consecutive 3-mm thick slices on the entire specimen were cut and stained with hematoxylin and eosin.

All thrombi specimens were in addition tested immunohistochemically for CD3 to detect and quantify CD3+ cells, i.e. T-cells, using an anti-human mouse monoclonal antibody (DakoCytomation code M7254, dilution 1/20).

Immunohistochemical slices were scanned and digitized with a slide scanner (Roche Ventana iScan Coreo). In some cases, the slide scanner failed to digitize slides, especially when CD3+ density was low. In these cases, manual scanning with the same magnification was performed, using a Leica system (Leica Microsystems, Switzer land, Leica EC3 digital color camera 3.1 Megapixel linked with Leica DM2500 microscope).

Histological examination was performed without knowledge of the clinical findings by a board-certified neuropathologist (V.R.) and a Ph.D. student (C.D.), and all slices on both standard coloration and immunohistochemical staining were analyzed.

Each immunohistochemical slide was analyzed at the same magnification (x200). Among all the slices observed on a given slide, the one with the highest number of CD3+ cells was chosen. Every slice present on each slide was observed before the high-density slice was chosen. A cell was considered positive for CD3+ marker when fulfilling 3 criteria: successful staining with cells clearly marked, compatible size, and visible nucleus. CD3+ T-cells were then manually counted, using the plugin Cellcounter available with the ImageJ software (ImageJ 1.48v, National Institutes of Health, USA).

### Statistical analysis

Baseline characteristics were summarized as means with standard deviation (SD), medians (with range) or frequency counts and proportions. A non-parametric Mann-Whitney was conducted to compare quantitative variables. A ROC curve and its Area Under Curve were estimated by Hanley’s method to define threshold of CD3+ discriminating “atherosclerotic” group. An ANOVA test was used to explore the association between blood parameters and immunohistochemical content. Statistical analysis was conducted using R software (version 3.1.1). Results were considered statistically significant if p-values were <0.05. Three groups were considered for the analysis: “atherothrombotic”, “cardioembolism”, and “other causes”.

## Results

### Clinical and Interventional Data

From January 2014 to September 2014, a total of 54 thrombi were retrieved from 54 patients with an average age of 64 years (±16). There were 34 men (63%) and 20 (37%) women.

Confirmed etiology was “cardioembolic” in 25 (46.3%) patients (24 atrial fibrillation and 1 flutter), “atherothrombotic” with artery-artery embolism from extracranial atherosclerosis in 10 (18.5%), and “other causes” in 19 (35.2%), including 3 (5.6%) thromboembolic complications during endovascular treatment of intracranial aneurysm, 1 (1.9%) Staphylococcus aureus acute infectious endocarditis, 3 (5.6%) thrombophilia (1 antithrombin III deficiency, 1 antiphospholipid syndrome, and 1 unknown thrombophilia in a 21 years old patient with medical history of several arterial and venous thrombosis), 2 (3.7%) arterial dissection, 1 (1.9%) intracranial stenosis, 3 (5.6%) undetermined (corresponding to one possible but not definitive cause, i.e. 3 permeable foramen ovale). Six (11.1%) strokes were of unknown cause despite complete exploration.

There were more men in the “atherothrombosis” group (9/10) in comparison to the cardioembolism group (male, 13/25), and “cardioembolism” and other causes group (male, 25/44).

Clinical characteristics including cardiovascular risk factors are summarized in Table 1.

**Table 1:** Patient characteristics and clinical data.

Age in years, mean (± SD)	64 ±16
**Sex (male), n (%)**	34 (63)
**Cardiovascular risk factors, n (%)**	
**Hypertension**	24 (44)
**Diabetes**	8 (15)
**Dyslipidemia**	21 (39)
**Smoking**	15 (28)
**Stroke etiology, n (%)**	
**Cardioembolic**	25 (46.3)
**Atherothrombosis (extracranial atherosclerosis)**	10 (18.5)
**Other causes**	19 (35.2)
**Thromboembolic complication during endovascular treatment**	3 (5.6)
**Thrombophilia**	3 (5.6)
**Arterial dissection**	2 (3.7)
**Intracranial stenosis**	1 (1.9)
**Endocarditis**	1 (1.9)
**Indeterminate**	3 (5.6)
**Unknown**	6 (11.1)
**Other**	4 (7.4)
**Baseline NIHSS, median (range)**	18 (8–26)
**Intravenous fibrinolysis (rtPA), n (%)**	31 (57)
**Site of arterial occlusion, n (%)**	
**Middle cerebral artery**	33 (61)
**Tandem occlusion**	9 (17)
**Carotid artery, terminal portion**	6 (11)
**Basilar artery**	5 (9)
**Cervical internal carotid**	1 (2)
**Thrombus orientation**	
** Maintained, n (%)**	26(48)
** Proximal**	9
**Central**	9
**Distal**	8
**Not maintained**	28 (52)

### Complete blood count (CBC)

Mean number of leukocytes was 9.4 ± 2.3 x 109/L (atherothrombosis group) and 8.9 ± 3.1x109/L (all other causes), p = 0.54. Eighteen patients (33%) had leukocytosis (white blood cells > 10x109/L), and no correlation between leukocytosis and higher NIHSS at admission was found (p = 0.6).

Mean number of lymphocytes was 1.86 ± 0.53x109/L in atherothrombosis group, 1.28 ± 0.47x109/L in cardioembolic group (p = 0.006), and 1.26 ± 0.5x109/L in other causes than atherothrombosis group, (p = 0.004). No patient presented with lymphocytosis (lymphocytes >4x109/L).

### Histopathological data

Out of the 54 thrombi, orientation was maintained for 26 (48%). Maintaining orientation of thrombus after extraction appeared easier for thrombi from cardioembolic sources (20/25, 80%) than for thrombi for atherothrombotic sources (6/10, 60%). On these 26 samples, immunohistochemical analysis was performed on the proximal part of the thrombus for 9, on the central part for 9, on the distal part for 8. For the 28 remaining, orientation was not registered due to clot presentation after retrieval.

Basic histological analysis (i.e. hematoxylin-eosin staining) was performed on the whole part of the thrombus for each case.

Finally, 138 hematoxylin-eosin slides were analyzed, with a minimum of 2 slices and a maximum of 35 slices by slide.

### Immunohistochemical results, univariate analysis (Tables 2 and 3)

**Fig 1:**
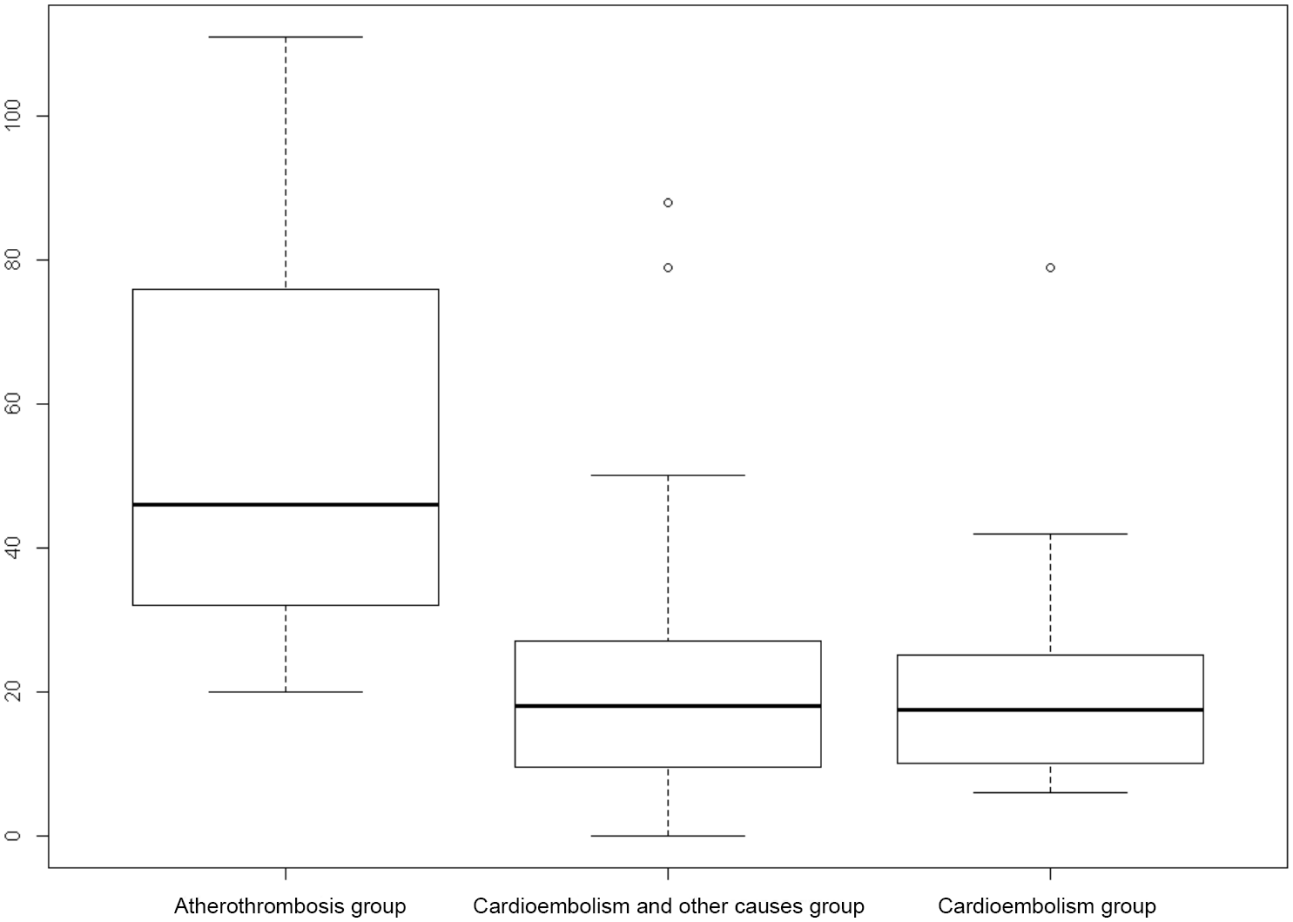
Boxplot showing distribution of CD3+ cells content in intracranial thrombi by stroke subtypes.

**Fig 2:**
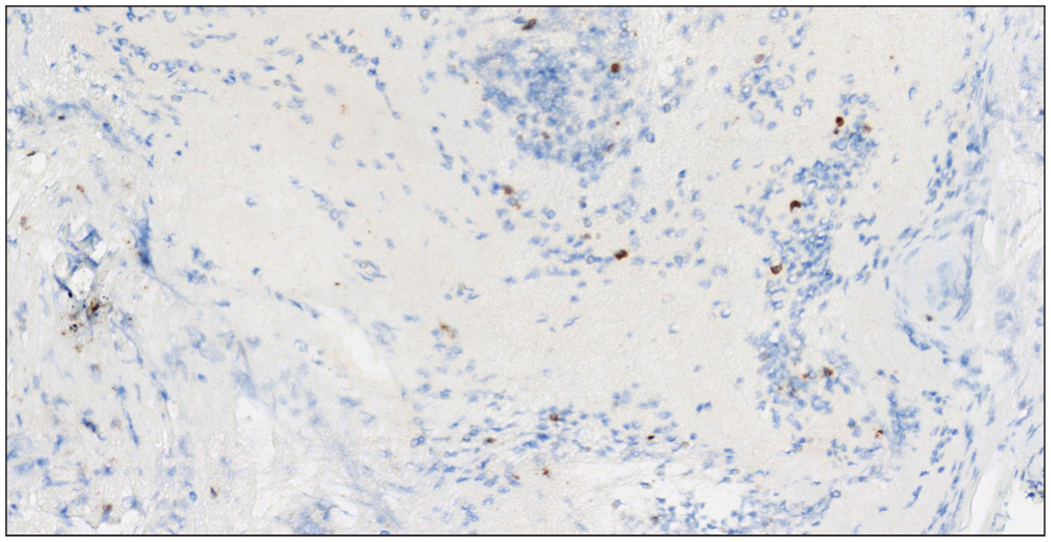
“Cardioembolic” thrombus. CD3+ cells (brown) corresponding to T cells in a “cardioembolic” thrombus.

**Fig 3:**
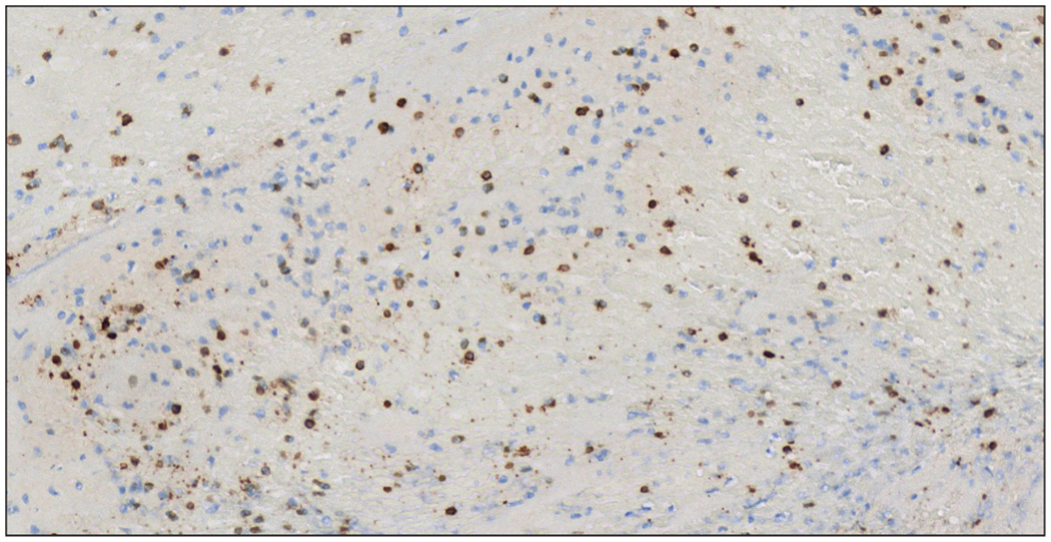
“Atherosclerotic” thrombus. CD3+ cells (brown) corresponding to T cells in an “atherosclerotic” thrombus.

There were significantly more CD3+ cells in thrombi from “atherothrombotic” group (53.60 ± 28.78) than in thrombi from “cardioembolic” (20.08 ± 15.66, p = 0.0003) group or “other causes” group (21.77 ± 18.31, p = 0.0005) (Fig 1, Tables 2 and 3, S1 File). Figs 2 and 3 give examples of T-cells in a “cardioembolic” thrombus and in an “atherosclerotic” thrombus.

**Table 2:** Number of CD3+ cells in “cardioembolism” and other causes thrombi and in “atherothrombosis” thrombi.

	All	“Cardioembolism” and other causes group (n = 44)	“Atherothrombosis” (n = 10)	p-value
Number of CD3+ cells	27.77 +/- 23.92	21.77 +/- 18.31	53.60 +/- 28.78	0.0005

**Table 3:** Number of CD3+ cells in “cardioembolism” thrombi and in “atherothrombosis” thrombi.

	All	“Cardioembolism” group (n = 25)	“Atherothrombosis” (n = 10)	p-value
Number of CD3+ cells	27.77 +/- 23.92	20.08 +/- 15.66	53.60 +/- 28.78	0.0003

CD3+ cells were randomly distributed in thrombi, and not restricted at their edges.

### Immunohistochemical results, multivariate analysis

After adjustment for percentage and number of lymphocytes on blood count, number of CD3+ cells was still higher in the “atherothrombotic” group (no significant interaction between number of CD3+ cells in thrombi and percentage or number of lymphocytes in CBC, p = 0.2).

### ROC curve

Threshold of 31.5 CD3+ cells per slice allowed discrimination of “atherothrombotic” thrombi with a sensibility of 0.80 (CI 95% 0.55–1) and a specificity of 0.81 (CI 0.70–0.93), a negative predictive value of 0.95 (0.93–1) and a positive predictive value of 0.5 (0.26–0.75).

### Thrombus morphology

Thrombi demonstrated different proportions of three components: fibrin/platelets, red blood cells and nucleated cells (either lymphocytes, monocytes macrophages and granulocytes).

**Fig 4:**
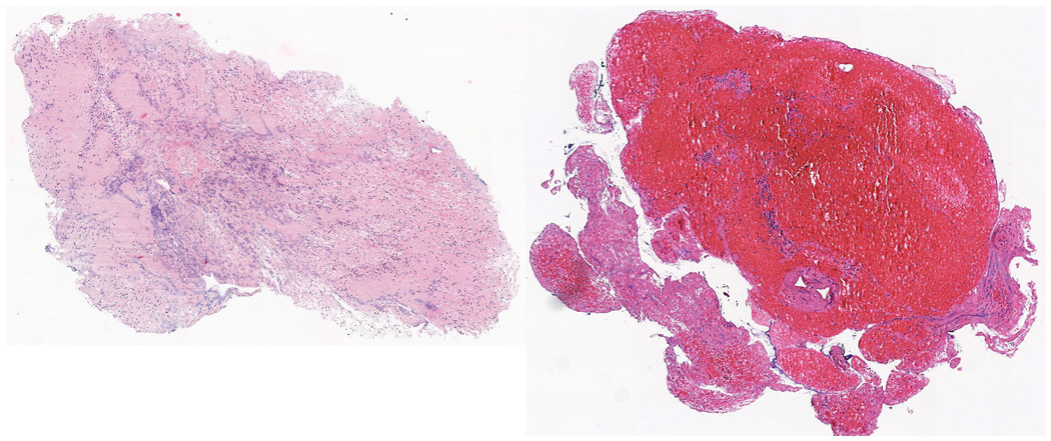
Hematoxylin and eosin-stained section of a fibrin-dominant thrombus (left) and of an erythrocyte-dominant thrombus (right).

At visual inspection, 14 (26%) were defined as fibrin dominant, 15 (28%) as red blood cells dominant, 25 (46%) as mixed. Fig 4 shows examples of fibrin-dominant and erythrocyte-dominant patterns.

No correlation was observed between thrombus morphology and stroke confirmed etiology.

## Discussion

We present an immunohistochemical study on intracranial thrombi, focusing on the analysis of CD3+ T-cells. To date, only a few histopathological studies on in-vivo intracranial thrombi have been reported in the literature [4,5,9,10,15–18], probably in part due to the obvious difficulties in obtaining and managing these kind of samples during an acute stroke case.

Although in our relatively small sample, the proportion of cryptogenic stroke was only 11%, in all patients presenting with stroke cryptogenic stroke accounts for 23% to 40% despite a complete workout, more frequently in younger patients [6]. This discrepancy may be due to the small sample size but also because patients with stroke due to large vessel occlusion have a higher proportion of cardioembolism and atherosclerosis than those without large vessel occlusion. The elucidation of stroke etiology may improve the secondary prophylactic therapy of stroke patients, and thus reducing stroke recurrence [19].

As observed in previous studies [4,5], a large diversity of histopathological pattern was found with standard coloration, but components were similar: fibrin and platelet bands interspersed with nucleated cells and erythrocytes [4,5,16]. These various patterns are probably reflecting different conditions of thrombus formation, regarding blood stream, shear stress and turbulences [4], but was not correlated to a specific etiology. However, a recent study [10] showed a higher proportion of red blood cells and a lower proportion of fibrin in clots from cardioembolism than with those from large-artery atherosclerosis. Thus, in contrast to previous studies, the study carried out by Kim and al. [10] supports the classical concept that cardioembolic thrombi forming in regions of stasis or slow flow are mainly composed of entrapped RBCs, whereas those occurring in the context of atherosclerotic large arteries are mainly composed of fibrin and platelets. We did not demonstrate a significant relation between red blood cell and fibrin composition and specific etiology, but this was not the study purpose. Especially, our division in red or white thrombi was based on macroscopic evaluation. We did not define a cut-off value to distinguish between red and white thrombi, as carried out by Niesten and al. [5]. We should note that maintaining orientation of the thrombus after extraction from the stent retriever appeared easier for thrombi from cardioembolic sources. This observation may reflect differences in mechanical properties, which are affected by the histologic characteristics of the thrombus [8].

Nevertheless, the aim of our study was to relate T-cells content in intracranial clot to stroke etiology. Our results highlight the importance of the number of CD3+ cells inside the thrombus retrieved after mechanical thrombectomy. We found significantly more CD3+ cells in thrombi from confirmed atherosclerotic causes compared to “cardioembolism” group and “other causes” group (p = 0.0005 and p = 0.0003). Furthermore, we demonstrated that these results were still significant after adjustment on CBC. In addition, thrombus orientation was maintained, in order to avoid biases potentially associated to an analysis focusing always on the same part of the thrombus. There is no data available on structural or composition difference on intracranial thrombus according to exposure to bloodstream or not, which led us to choose to equilibrate analysis of proximal, middle and distal parts on our samples.

There is limited data also available concerning leukocytes in intracranial thrombi retrieved from patients with acute stroke. Initial studies on this topic reported leucocytes present in small amounts, but without any specific analysis or quantification. One recent study [9] found that a higher percentage of white blood cells in the thrombus was associated with 3 clinical variables: cardioembolic etiology, extended mechanical recanalization time, and less favorable recanalization and clinical outcome. Nevertheless, the authors only analyzed white blood cells as a whole (T and B lymphocytes, granulocytes, monocytes/macrophages), potentially reducing the accuracy of the results.

Because of the lack of data, it is hard to put forward hypotheses concerning the role of various leukocytes populations in intracranial thrombus formation. Furthermore, studies carried out on inflammatory cells and cerebrovascular complications of atherosclerotic disease, have only focused on carotid plaques, not on intracranial thrombi. Nevertheless, we should note that the cardiology literature suggests an important role for leukocytes in thrombus growth [20].

By contrast, the role of leukocytes, especially macrophages and T-cells, is more clearly defined in atherosclerotic disease, especially in vulnerable lesions [21]. The final step of the disease is plaque destabilization and rupture, induced by the release of metalloproteinases and elastases into the fibrous cap, by the activated leukocytes (including CD3+) accumulating locally [22]. It has been showed that inflammatory components in internal carotid artery atherosclerotic plaque were correlated to the occurrence of ischemic events [23]. Furthermore, number of CD3+ cells in complicated plaques is higher than in uncomplicated ones [12]. In another study, ruptured plaques of patients affected by stroke were characterized by the presence of a more severe inflammatory infiltrate compared to plaques observed in the transient ischemic attack and asymptomatic groups [24]. Similarly, it has been shown that an immune-inflammatory cell accumulation accompanies the early stages of coronary atherogenesis, characterized by T-lymphocyte accumulation, both in the intima and adventitia [25]. Neutrophils have also a role in the formation, stabilization and growth of peripheral and coronary thrombi, especially through their interaction with activated platelets [26,27].

On this basis, we assumed that this complex inflammatory process occurring in atherothrombotic lesions might also influence the intracranial thrombus composition especially regarding the CD3+ ratio.

Interestingly, CD3+ T-cells in intracranial thrombi were distributed randomly inside the thrombi, and not restricted on their edges, suggesting that CD3+ cells are included during the initial phase of thrombus formation of the atherosclerotic lesion.

Despite our study being based on the largest sample of in-vivo intracranial thrombi available in the literature, the number of patients is still very limited and further investigations are mandatory to confirm our findings. Furthermore, other sub-populations of T-cells (CD4+ and CD8+) were not studied and may offer additional information for future identification of stroke etiology. Peripheral lymphocyte count was higher in the atherothrombosis group, but this finding was not significant enough to consider it as a biomarker of atherothrombotic stroke, given the several cell types composing this count (B- and T-cells and their subsets). We should note that a recent study has shown that subjects with cardioembolic stroke had a significantly higher peripheral frequency of CD4+ cells and CD28 null cells (a subtype of T-cells) compared to subjects with other TOAST subtypes [28].

There are two other aspects that merit consideration. First, our histological biomarker (CD3+) has been established and tested on the same cohort, limiting its validity. However, this approach was conducted given the difficulties to obtain human thrombi in a reasonable number during the study period. Second, although manual counting on each slice provided reproducibility regarding the identification of T-cells, this approach remained semi-quantitative. Further studies should consider more advanced techniques like fluorescence-activated cell sorting (FACS) in order to better assess T-cells content of intracranial thrombi. However, it is known that the fixation process may alter sample integrity, and this limit should be considered with thrombi analyzed with FACS [29].

Overall, we think that immunochemistry may be helpful to characterize T-cell content in cerebral thrombi. Immunochemistry may also demonstrate other components of human thrombi, as proteins implicated in inflammation processes and atherosclerosis. Recently, high mobility group box 1 (HMGB1) cytokine, a nuclear protein that regulates gene expression and acts as a pro-inflammatory alarmin, has been identified in human atherosclerotic plaques [30]. This protein is highly expressed in human coronary artery thrombi as well, and its binding to activated platelets could indicate a possible role for platelets/HMGB1 interactions in the physiopathology of atherothrombosis [31]. Therefore, HMGB1 could be studied in human cerebral artery thrombi by immunochemistry, in order to assess both its presence and whether its expression level varies with stroke etiology or not.

## Conclusion

Endovascular treatment of stroke and specifically successful clot retrieval allows now the analysis of thrombi previously inaccessible, giving rise to better knowledge and insight into the pathophysiology of stroke.

We present the first immunohistochemical study focusing on CD3+ T-cell content in intracranial thrombi retrieved from patients with large vessel stroke. Thrombi of “atherosclerotic” origin demonstrate significantly more CD3+ cells than the other groups.

Hence, this study suggests that high proportions of CD3+ cells in intracranial thrombi may be a reliable biomarker for stroke of atherothrombotic origin. This finding needs to be validated in larger studies.

## Supporting Information

S1 File CD3+ count. (XLSX)
